# Preferences for Subtyping Primary Aldosteronism: A Discrete Choice Experiment

**DOI:** 10.1210/jendso/bvaf185

**Published:** 2025-11-22

**Authors:** Maame Esi Woode, Winston Chong, Elisabeth Ng, Shanshan Lu-Shirzad, Stella May Gwini, Peter J Fuller, Jun Yang, Gang Chen

**Affiliations:** Centre for Health Economics, Monash Business School, Monash University, Caulfield East, VIC 3145, Australia; Victorian Heart Institute, Monash University, Clayton, VIC 3168, Australia; Alfred Health Radiology, Alfred Health, Melbourne, VIC 3004, Australia; School of Translational Medicine, Faculty of Medicine, Nursing and Health Sciences, Monash University, Melbourne, VIC 3004, Australia; Centre for Endocrinology and Reproductive Health, Hudson Institute of Medical Research, Clayton, VIC 3168, Australia; Centre for Endocrinology and Reproductive Health, Hudson Institute of Medical Research, Clayton, VIC 3168, Australia; Centre for Endocrinology and Reproductive Health, Hudson Institute of Medical Research, Clayton, VIC 3168, Australia; School of Public Health and Preventive Medicine, Monash University, Melbourne, VIC 3004, Australia; Centre for Endocrinology and Reproductive Health, Hudson Institute of Medical Research, Clayton, VIC 3168, Australia; Centre for Endocrinology and Reproductive Health, Hudson Institute of Medical Research, Clayton, VIC 3168, Australia; Department of Medicine, Monash University, Clayton, VIC 3168, Australia; Centre for Health Economics, Monash Business School, Monash University, Caulfield East, VIC 3145, Australia; Centre for Health Policy, Melbourne School of Population and Global Health, University of Melbourne, Melbourne, VIC 3010, Australia

**Keywords:** primary aldosteronism, hypertension, subtyping, predictive algorithm, PET-CT, adrenal vein sampling

## Abstract

**Context:**

Primary aldosteronism (PA) affects 10% to 15% of individuals with hypertension and increases cardiovascular risk. Differentiating between unilateral and bilateral PA determines optimal treatment and typically requires adrenal vein sampling (AVS). Emerging subtyping methods include predictive algorithms and nuclear imaging.

**Objective:**

This study explores hypertensive individuals' preferences for different PA subtyping strategies.

**Methods:**

Two labeled discrete choice experiments (DCEs) evaluated preferences for subtyping methods based on test accuracy, waiting time, adverse effects, and out-of-pocket cost. Latent class conditional logit (LCL) modeling segmented participants by preferences, while policy simulation analyses examined uptake variations by age, sex, and income.

**Results:**

Among 583 hypertensive Australian adults (mean age: 48 years; 48% female), 85% were willing to undergo PA subtyping. Participants prioritized accuracy, shorter waiting times, minimal side effects, and lower costs. LCL analysis revealed that participants who were older, female, or considered themselves less busy were more likely to opt for PA subtyping. Subtyping uptake was highest for algorithm-based methods (∼54%) with its uptake rate increasing to 68% after factoring in cost.

**Conclusion:**

Preferences for PA subtyping are driven by cost, invasiveness, and waiting time. Less-invasive, faster, and low-cost methods were preferred, even if they are slightly less accurate than AVS. Further research is needed to optimize the accuracy of subtyping algorithms and facilitate implementation in clinical practice.

Primary aldosteronism (PA) is a substantial cause of hypertension [1]. It is characterized by the autonomous secretion of aldosterone by one or both adrenal glands, leading to sodium retention, high blood pressure [2], and an increased risk of cardiovascular disease [3]. A recent study in Australia found the prevalence of PA in patients with treatment-naive hypertension in the primary health-care setting to be 14% [1].

Diagnosing PA involves screening with a blood test for the aldosterone-to-renin ratio, followed by confirmatory testing if this ratio is elevated above a set threshold [4]. Once PA is confirmed, an adrenal computed tomography (CT) scan and adrenal vein sampling (AVS) are performed to subtype the disease as either unilateral or bilateral. Unilateral PA can be cured with surgery to remove the adrenal gland that is producing excess aldosterone, while bilateral PA requires long-term targeted medical therapy with mineralocorticoid receptor antagonists [2, 5, 6].

AVS, in which blood is collected from both adrenal veins to measure aldosterone levels, is currently considered the gold standard for subtyping PA [2, 5, 7, 8]. This test is, however, invasive, technically challenging, time-consuming (taking up to 3 hours for the procedure and up to 1 day for preparation and rest time), costly, and performed at few specialist centers [5, 7]. Two potential alternatives for subtyping being explored are functional imaging using positron emission tomography–CT (PET-CT) [9] and the use of a predictive algorithm [5, 7]. PET-CT imaging involves the administration of a radioactive tracer into the blood to allow detection of aldosterone-producing adenomas; this can take more than 30 minutes depending on the tracer/ligand [9]. This procedure is less invasive and less time-consuming compared to AVS. It is currently available only in specialist centers for research use. A substantially less expensive and less time-consuming option previously proposed is the use of a predictive algorithm [5, 7]. This involves using readily available patient information in a validated algorithm to predict the likelihood of unilateral or bilateral disease.

This study aimed to explore the community's preferences for different PA subtyping procedures, that is AVS, PET-CT, and a predictive algorithm. Additionally, simulation analyses were conducted to estimate uptake rates for the 3 subtyping strategies under different hypothetical scenarios. Findings from this study will facilitate the better implementation of subtyping for PA in the health-care system.

## Materials and Methods

### Study Design

A discrete choice experiment (DCE) was used to investigate preferences for the use of AVS, PET-CT, and a predictive algorithm in PA subtyping.

### Development of the Discrete Choice Experiment

#### Attributes and levels

To decide on attributes (characteristics) and levels that are most likely to influence a person's choice of subtyping strategy, a literature search was conducted, followed by interviews with clinical experts and consumers. This yielded a list of 7 potential attributes: surgical success based on biochemical outcomes, surgical success based on clinical outcomes, waiting time, invasiveness of test procedure, potential side effects, influence on quality of life, and out-of-pocket costs.

Further discussion with the research team narrowed down the list of attributes to 4. Surgical success based on biochemical outcomes was considered more relevant to consumers, hence it was kept in the list as subtyping accuracy, while clinical success was removed. Subtyping accuracy was subsequently defined as the probability of the test giving an accurate diagnosis of surgically curable disease (expressed as, eg, “70% likely to give an accurate diagnosis of surgically curable disease”). Levels for PET-CT [10-14] and algorithmic [15, 16] subtyping accuracy were obtained from the literature, expert opinion, and local experience. The level of invasiveness was entirely dependent on the subtyping procedure; hence it was removed from the list and incorporated into the introduction to the subtyping strategies. The influence on quality of life was directly related to side effects; hence both were considered within the side effects attribute. The out-of-pocket cost levels were determined through consultation with consumers via the “Conn's Syndrome/Hyperaldosteronism Support Group” on Facebook (https://www.facebook.com/groups/652067311558303/), and clinical experts who routinely manage PA. The final list of 4 attributes selected as relevant for consumers were surgical success, waiting time for the test, side effects, and out-of-pocket costs.

As out-of-pocket costs were likely to have a strong influence on consumer choice, we designed 2 experiments (to be introduced in detail in the next section), one with and another without out-of-pocket costs attached. The experiment without mention of direct out-of-pocket costs mimics the situation at public hospitals where costs are covered through Medicare, a universal health care insurance program for Australian residents. The model with costs is more aligned with services provided in the private health sector.

The initial survey was pretested with 3 patients who had either gone through AVS or were considering it. They did not identify other relevant attributes but suggested revisions to the definition and the levels of the attributes and to the introductory information provided on PA subtyping. These changes were implemented where possible and a revised version of the questionnaire was provided to them for final comments. The final attributes and levels are presented in Table 1.

**Table 1.** The final attributes and their respective levels for each attribute used in the designing of the discrete choice experiment*^a^*

Attribute	Algorithm	AVS	PET-CT
**Subtyping accuracy** (likelihood of accurate diagnosis of surgically curable disease)	−70% −80%	−70% −80% −90% −95%	−70% −80% −90% −95%
**Waiting time for procedure**	−None −2 weeks	−2 weeks −3 months −6 months −12 months	−2 weeks −3 months −6 months −12 months
**Side effects**	−None	−None −Minor (eg, swelling, bleeding, or bruising at injection site) −Moderate (eg, adrenal gland bleeding [very rare], allergic reaction) −Serious (eg, deep vein thrombosis (clot) [very rare])	−None −Minor (eg, swelling, bleeding, or bruising at injection site) −Moderate (eg, allergic reaction)
**Out-of-pocket costs**	−$0	−$0 −$250 −$500 −$1000 −$1500	−$0 −$250 −$500 −$1000 −$1500

Abbreviations: AVS, adrenal vein sampling; PET-CT, positron emission tomography–computed tomography.

^a^Experiment 1 has only 3 attributes, namely subtyping accuracy, waiting time for procedure, and side effects, whereas experiment 2 has all 4 attributes.

#### Experimental design

Two labeled DCEs were designed for this study, one without the cost attribute and the other with all the attributes included. We focused on the main effects model and generated a D-efficient design with zero priors (assuming no prior knowledge about participants' preferences) on Ngene v1.2.1 DCE design software (ChoiceMetrics). This design focuses on the individual effect of the attributes (main effects) without modeling interactions between attributes. For each DCE design, 1 with a cost attribute and 1 without the cost attribute, a total of 60 choice sets/tasks (Supplementary Fig. S1 [17]) were generated that were further divided into 12 versions, so that each participant answered 5 choice tasks per DCE type. In total, each participant therefore faced 10 choice tasks. The order in which the 2 DCEs were presented to each participant was randomized.

Participants were first asked to select between AVS, algorithm, and PET-CT. As a follow-up question, participants were requested to indicate whether their choice would remain the same if they had an option to opt out of the subtyping test they preferred. An example of a choice set faced by a participant is presented in Supplementary Fig. S1 [17].

### Online Survey

The final survey was hosted online via the Qualtrics platform (www.qualtrics.com). The first section of the survey focused on the choice experiment. To assess participant rational decision-making and understanding of the choice experiment, a dominant choice task was included prior to the 10 DCE tasks. This pairwise choice task presented 2 screening services, in which 1 option was better (dominant) than the other, with this dominant service expected to be chosen by means of a rational decision-making process. On failing to choose the dominant task, participants were provided information on what their choice meant and then given a second chance to select the dominant task (where the left-right position of the 2 tests was switched). Regardless of whether participants failed or passed the dominant task, they were presented with the 10 actual DCE tasks afterward.

After completing the DCE, the following sections collected information relating to participants' demographic and socioeconomic characteristics (eg, pre-tax or gross household income in the past year), their hypertension, PA, and AVS knowledge, and their general health and quality of life.

### Participants

The aim was to recruit 550 participants from the Australian general public who self-reported having high blood pressure or who were taking medication to control their blood pressure at the time of the survey. Participants were recruited by Dynata (www.dynata.com), one of the world's largest first-party data platforms, between December 20, 2023, and January 11, 2024.

### Statistical Analyses

#### Preferences for subtyping

The theoretical foundations of DCE are based on random utility theory. Based on this theory, the utility ($U_{nst}$) that participant *n* gains from choosing subtyping procedure *s* in choice situation *t* can be expressed as:


(1) $$U_{nst}=\beta^{\prime }x_{nst}+\varepsilon_{nst}\;\;\;$$

where $x_{nst}$ denotes the vector of observed attributes (ie, subtyping test characteristics—accuracy, waiting times, side effects, and costs) while *β* denotes the vector of preference parameters to be estimated (coefficients of interest). The higher the coefficient relative to the reference category, the higher the preference for that level of the attribute. $\varepsilon_{nst}$ refers to the random component assumed to be independently and identically distributed. This captures any unobserved factors that influence the participant's utility.

Assuming a homogeneous preference among participants, we used a conditional logit model to estimate preferences. In reality, not all individuals value the attributes in the same way, that is, their preferences are heterogeneous. Therefore, we further ran a latent class conditional logit (LCL) model, which assumes the presence of subgroups (latent classes) of individuals with different preference patterns. This allowed us to look for groupings in preferences based on participant characteristics (eg, age). To explore the latent class subgroups, we considered participants' age, sex, income, knowledge and experience with PA, time availability, and effects of high blood pressure on participants. The number of latent classes to retain was decided based on the Bayesian information criterion (with the lower values indicating more preferred models). The DCE data were analyzed using NLOGIT software.

Our model was run on a main study sample that excluded participants who either (1) failed the dominant choice tasks (ie, chose irrationally, such as ignoring an obviously superior option), or (2) completed the whole survey in less than one-third of the median duration of all participants (ie, those who completed the survey too quickly, suggesting a quality issue). We also ran the conditional logit model on a sample that excluded only those who spent less than one-third of the survey's median duration for robustness analyses (full sample results in Supplementary Table S1 [17]).

#### Policy simulation: subtyping procedure uptake rates

Based on the estimated coefficients from the DCE data, we could predict the potential uptake rates of different subtyping procedures described using the attributes and levels in the experiment. A base case scenario was created based on the most probable attribute level for each subtyping procedure using expert opinion (Table 2). The attribute levels for AVS and PET-CT were set to be equivalent to each other with the algorithm set to its best attribute levels. This implied that the accuracy of the algorithm was lower than the accuracy for both AVS and PET-CT. The main hypothesis here was that if accuracy was more important, then participants would generally choose between AVS or PET-CT, both of which provide the same level of accuracy, bearing in mind that the levels of invasiveness of the procedure and waiting time were also under consideration, with participants able to opt out if they preferred not to proceed to subtyping.

**Table 2.** Base case attribute levels used in estimating uptake rates and for policy simulations

Attribute	AVS	Algorithm	PET
Accuracy	90% likely to give accurate diagnosis of surgically curable disease	80% likely to give accurate diagnosis of surgically curable disease	90% likely to give accurate diagnosis of surgically curable disease
Waiting time	3 months	None	3 months
Side effects	Minor	None	Minor
Cost	AU$500	AU$0	AU $500

Abbreviations: AVS, adrenal vein sampling; PET-CT, positron emission tomography–computed tomography.

We further ran several simulations by carrying out specific subgroup analyses based on the participants' household income, sex, and age. The latter 2 demographic characteristics have been highlighted in the literature on the uptake of cardiovascular prevention [18], while the household income level could influence participants' willingness to pay for the subtyping test if an out-of-pocket cost is required, particularly for low-income people (who may be more price-sensitive).

## Results

### Characteristics of Participants

A total of 653 participants with high blood pressure completed the survey. Of these, 70 were excluded from the main sample due to either failing the dominant choice task or completing the survey too quickly (in <4 minutes, less than one-third of the median time). This left a main analysis sample of 583 participants; an expanded “full sample” of 637 was also used for sensitivity analyses, which excluded only those who completed the survey too quickly.

The main sample had an average age of 48 years and a slight female majority (50.6%) (Table 3). Most participants were born in Australia (79.1%), and 53.1% had an annual household income of Australian dollars (AU$)80 000 or less, slightly skewed compared to the national median income of AU$93 028 (2019-2020 data). While 39.8% of participants had heard of PA, only 17.3% reported understanding the condition. In total, 15.6% reported they were either currently receiving treatment for PA or had undergone surgery for it. The remaining 84.4% either had not heard of PA, had heard of it but were not receiving treatment, or were unsure.

**Table 3.** Description of the sample population used in the discrete choice experiment. Presenting the characteristics of both the main sample (excluding those who answered survey too quickly and those who failed the rationality test) and the full sample (excluding only those who answered survey too quickly)

	Main sample		Full sample	
Variable	N	Value	N	Value
**Age (mean (SD)), y**	583	48.3 (17.6)	637	47.1 (17.7)
Sex				
Male	282	48.4%	311	48.8%
Female	295	50.6%	319	50.1%
Other	5	0.9%	6	0.9%
Prefer not to say	1	0.2%	1	0.2%
State				
Australian Capital Territory	18	3.1%	19	3.0%
New South Wales	170	29.2%	192	30.1%
Northern Territory	2	0.3%	2	0.3%
Queensland	104	17.8%	112	17.6%
South Australia	59	10.1%	60	9.4%
Tasmania	25	4.3%	26	4.1%
Victoria	155	26.6%	174	27.3%
Western Australia	50	8.6%	52	8.2%
Born in Australia				
Yes	461	79.1%	509	79.9%
No	122	20.9%	128	20.1%
Highest level of education				
≤Primary school	5	0.9%	6	0.9%
Secondary school	159	27.3%	177	27.8%
Trade certificate/Vocational	146	25.0%	156	24.5%
Undergraduate degree	210	34.5%	216	33.9%
Postgraduate degree	70	12.0%	79	12.4%
Don’t know/Unsure	2	0.3%	3	0.5%
Income				
≤$40 000	110	18.9%	120	18.8%
$40 001-$80 000	202	34.7%	218	34.2%
$80 001-$120 000	124	21.3%	133	20.9%
$120 001-$160 000	66	11.3%	75	11.8%
$160 001-$200 000	34	5.8%	40	6.3%
≥$200 001	27	4.6%	29	4.6%
Don't know/Prefer not to say	20	3.4%	22	3.5%
Have you heard of PA?				
Yes, heard of it and understand it	101	17.3%	133	20.9%
Yes, heard of it but no understanding	131	22.5%	143	22.5%
No	333	57.1%	340	53.4%
Not sure	18	3.1%	21	3.3%
Do you have PA?				
Yes, receiving treatment	64	11.0%	83	13.0%
Yes, has had surgery	27	4.6%	42	6.6%
No or have not heard of PA	430	73.8%	445	69.9%
Not sure	62	10.6%	67	10.5%
Too busy to worry about BP				
Yes	115	19.7%	139	21.8%
No	468	80.3%	498	78.8%
HBP doesn’t make me unwell				
Yes	287	49.2%	313	49.1%
No	296	50.8%	324	50.9%

Abbreviations: BP, blood pressure; HBP, high blood pressure; PA, primary aldosteronism.

Around 20% of participants indicated they were too busy to worry about their blood pressure, while nearly half (49.2%) reported that their blood pressure did not make them feel unwell. Still, 96.5% considered high blood pressure to be a serious issue. Notably, 84.9% of participants were open to undergoing further testing if there was a possibility their blood pressure had a curable cause. Approximately 23.5% had previously been hospitalized due to high blood pressure, yet 78.7% reported being currently satisfied with their blood pressure management.

### Findings From the Discrete Choice Experiment

#### Preferences for primary aldosteronism subtyping procedures

**Figure 1.**
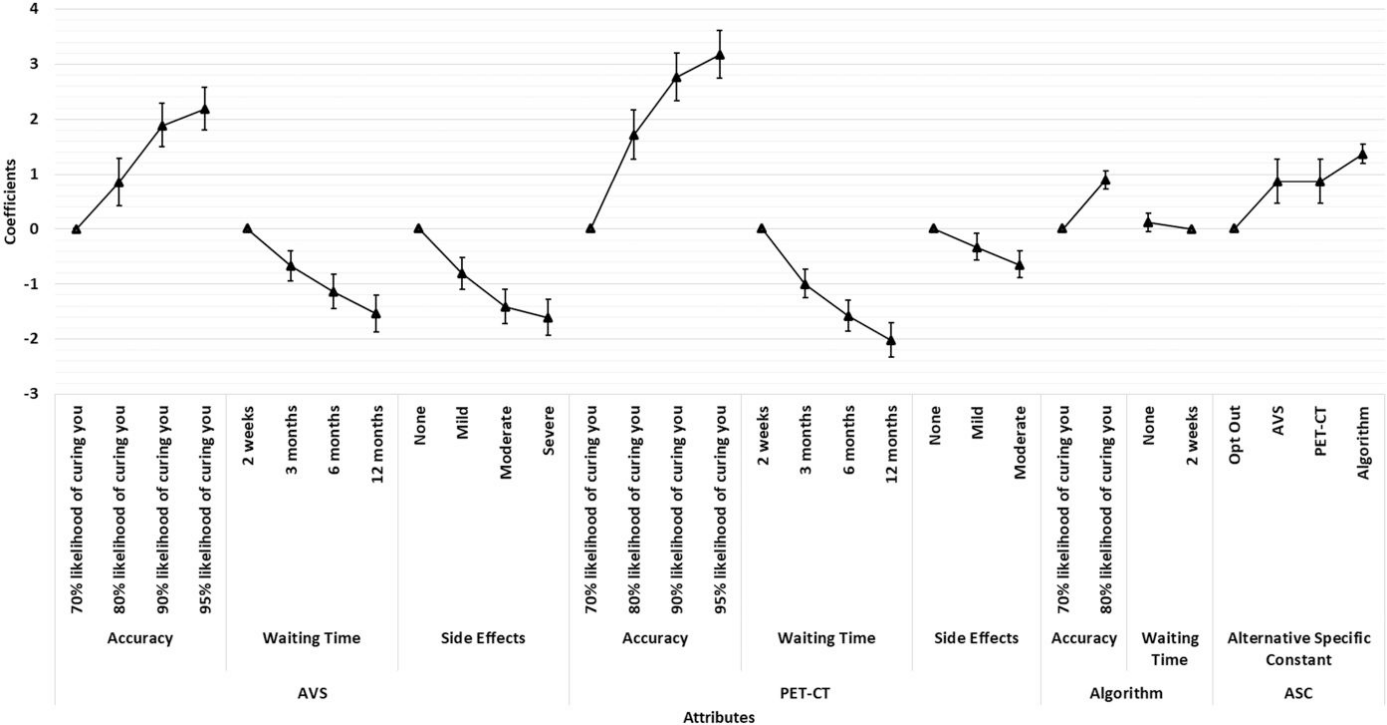
Preferences for primary aldosteronism subtyping in the absence of costs, with each triangle representing estimated coefficients for each attribute with 95% confidence intervals (CIs) presented as vertical lines. Coefficients are compared within each attribute; thus, the higher the coefficient of a level within an attribute, the higher the preferences for the level within that attribute. Abbreviations: ASC, alternative specific constant; AU, Australian; AVS, adrenal vein sampling; PET-CT, positron emission tomography–computed tomography.

**Figure 2.**
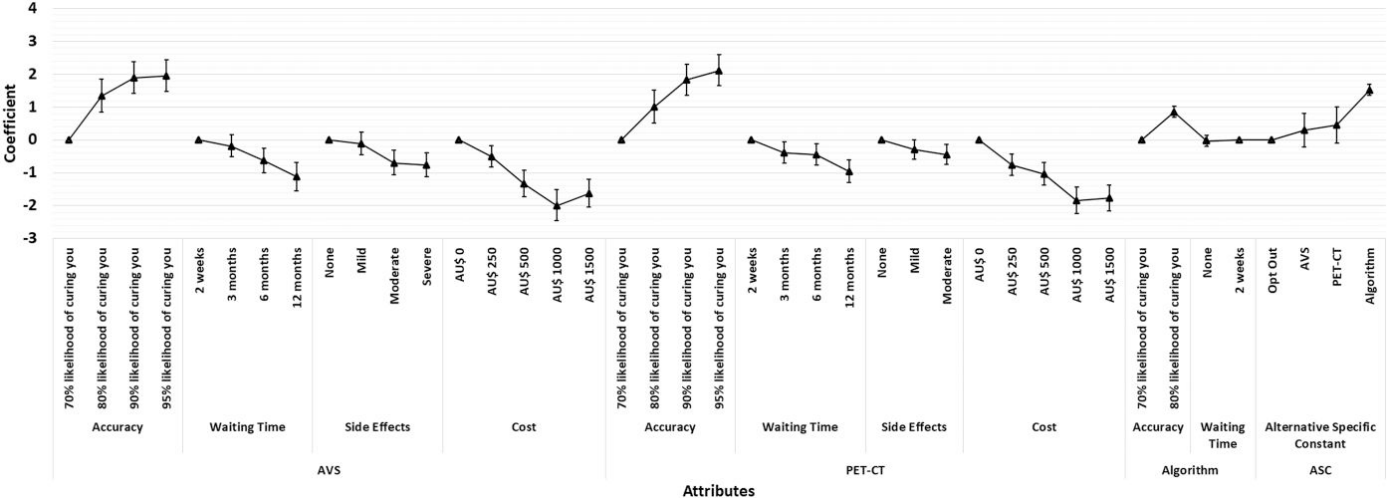
Preferences for primary aldosteronism subtyping with the inclusion of costs, with each triangle representing estimated coefficients for each attribute with 95% CIs presented as vertical lines. Coefficients are compared within each attribute; thus, the higher the coefficient of a level within an attribute, the higher the preferences for the level within that attribute. Abbreviations: ASC, alternative specific constant; AVS, adrenal vein sampling; PET-CT, positron emission tomography–computed tomography.

The main findings from the DCE are summarized in Figs. 1 and 2 (with full results in Supplementary Table S1 [17]). Participants consistently preferred subtyping strategies that were more accurate, had shorter waiting times, and carried fewer side effects. These preferences were observed regardless of whether out-of-pocket costs were included. There was a general preference both for the algorithm and AVS compared to opting out of subtyping. PET-CT was also viewed positively, though its preference was not statistically significant in all models.

When out-of-pocket costs were introduced (up to AU$1500), preferences shifted toward less costly options. The preference for AVS over no subtyping became nonstatistically significant at higher cost levels, while the algorithm and PET-CT remained preferred. These results were consistent in the full sample.

#### Differences in preferences across participant subgroups

LCL analysis revealed 2 distinct groups based on preference patterns based on the Bayesian information criterion. In the version of the DCE without cost information, 60% of participants (class 1) showed strong interest in subtyping, whereas the remaining 40% (class 2) was less engaged, particularly with algorithms, when all other factors were held constant.

Older adults (age ≥ 50) were more likely to belong to class 1, while men, individuals who had received PA treatment, and those who reported being “too busy to worry” about blood pressure, were more likely to be in class 2.

When cost was included, class 1 comprised 66% of participants and class 2 comprised 34%. In class 2, PET-CT was viewed less favorably, and neither the side effects of AVS nor the waiting time for PET-CT significantly influenced decisions. Across both groups, lower costs were consistently preferred. Full model estimates and subgroup characteristics are provided in Supplementary Tables S2 [17] and S3 [17].

#### Policy simulation results

Using our base case scenario (see Table 2), we simulated how preferences would change across subgroups if out-of-pocket costs were introduced. Across age, sex, and income, the predictive algorithm had the highest uptake followed by PET-CT and then AVS, reflecting preferences for lower invasiveness and shorter duration.

**Figure 3.**
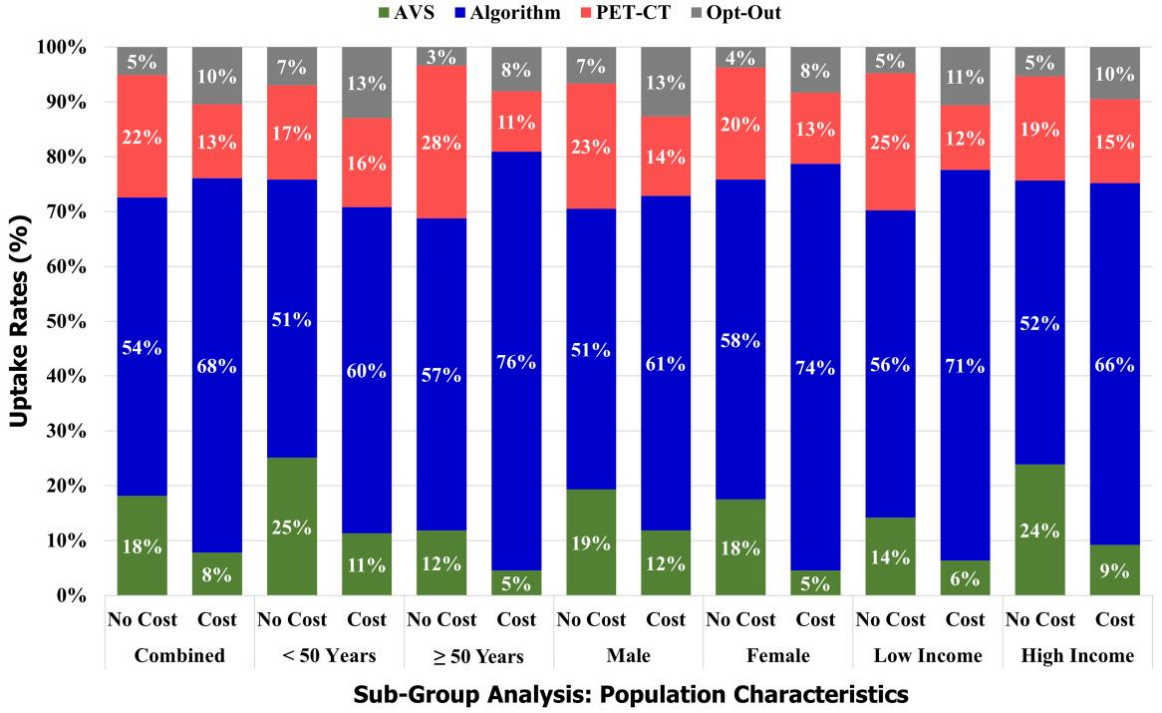
Subgroup analysis of subtyping uptake rates based on age, sex, and socioeconomic status. AVS, adrenal vein sampling; PET-CT, positron emission tomography–computed tomography.

Younger adults (age <50 years) and low-income participants preferred AVS more than their older or higher-income counterparts, particularly when no cost was involved. However, across all groups, out-of-pocket costs reduced uptake both of AVS and PET-CT and increased interest in algorithms and opting out altogether (Fig. 3).

Older adults were more likely to choose the algorithm than younger adults (eg, 76% vs 60% when costs were included), and less likely to opt out of subtyping. Similarly, women were more likely than men to choose the algorithm (74% vs 61% with costs) and less likely to decline subtyping altogether. The full preference coefficients for attributes for each policy simulation can be found in the supplementary materials [17] (Supplementary Tables S4 [17] to S6 [17]).

Cost had a clear deterrent effect (Table 4). Introducing out-of-pocket costs increased opt-out rates by 77% to 140% across groups and increased the uptake of algorithms by about 17% to 27%. The decline in AVS and PET-CT use was more pronounced for AVS, particularly in the high-income group (61% drop vs 55% in the low-income group), among women (73% drop vs 39% drop for men), and in the older age group (62% vs 55% for older age groups). PET-CT uptake fell more steeply among low-income participants (53% vs 19%) and older participants (60% vs 5%).

**Table 4.** Subgroup analysis (age, gender and level of household income) of changes in uptake rates due to the introduction of costs*^a^*

Method	Main sample	<50 y	≥50 y	Male	Female	Low income	High income
AVS	−57%	−55%	−62%	−39%	−74%	−55%	−61%
Algorithm	25%	17%	34%	19%	27%	27%	27%
PET-CT	−40%	−5%	−60%	−37%	−37%	−53%	−19%
Opt-Out	104%	85%	140%	91%	125%	124%	77%

Abbreviations: AVS, adrenal vein sampling; PET-CT, positron emission tomography–computed tomography.

^*a*^Each percentage is defined as follows: $((Uptake\;Rates_{Costs}-\;Uptake\;Rates_{No\;Costs})/Uptake\;Rates_{No\;Costs})*100$

## Discussion

This is the first study to investigate the preferences for PA subtyping strategies from the consumers' perspective. The main goal was to investigate how patients value trade-offs between the burdens, risks, and benefits associated with the 3 subtyping strategies. The study did not aim to compare the clinical diagnostic utility of each modality.

The key findings indicate that noninvasive and time-efficient strategies are preferred irrespective of income group, age, and sex. Unique to the health preference study, by directly administrating 2 DCE (with vs without out-of-pocket costs) and comparing their preferences, we quantified the influence of the financial burden on participants' subtyping preference. Participants were price-sensitive with the effect being much stronger for the low-income group vs the high-income group. Furthermore, in the Australian context, where both public and private health services exist, charging out-of-pocket costs for AVS and PET-CT was predicted to have more people choosing the cost-free predictive algorithms if these options are available. With the limited availability and variable success of AVS, and emerging subtyping strategies using functional PET-CT imaging or predictive algorithms, these results crucially inform clinicians and policymakers of the patient perspective on each strategy [5, 7].

This study focused on 3 subtyping strategies, namely AVS, PET-CT, and predictive algorithms. Four attributes were considered in the experiment: accuracy, waiting time for results, potential side effects of the subtyping procedure, and costs. Overall, there was a general preference for PA subtyping as opposed to opting out of any subtyping. Results indicated substantial interest in subtyping using less-invasive procedures that required less time, and with fewer side effects. While accuracy was an important determinant of willingness to subtype, we found that participants were willing to go through subtyping with a strategy that is slightly less accurate if it required limited to no invasive procedures, and that had no potential side effects or extra costs. Uptake rates were highest for predictive algorithms as they possessed all these features valued by patients.

The current gold-standard subtyping procedure, AVS, was preferred over either PET-CT or the predictive algorithm when it achieved better success rates relative to the other alternatives, with similar or better waiting times, and side effects. This emphasizes the need for centers that offer AVS to ensure consistently high performance to justify offering the service to patients who choose to undergo subtyping. This is particularly important given the highly variable rates of AVS success [19].

Functional imaging with PET-CT is a rapidly evolving field with novel radiotracers used for the lateralization of aldosterone-producing adrenal adenomas [20, 21]. At present, PET-CT is available only within the research setting, but our results indicate that, if offered, consumers would choose it over AVS should it offer equal accuracy, waiting time, and risk of side effects. Consumer choice may, however, be limited by the clinical scenario and availability of infrastructure for functional imaging. A health economic evaluation may also be warranted before the wider use of PET-CT for subtyping.

Subtyping using predictive algorithms was the preferred strategy by the majority of consumers when accuracy levels were at 80% compared to up to 90% for the other subtyping alternatives, based on the desirable attributes of being quick, noninvasive, and free of side effects or cost. However, the accuracy of an algorithm for identifying surgically curable PA is highly variable. In addition, the proportion of patients who can benefit from the algorithm decreases with higher levels of accuracy (ie, higher specificity of an algorithm for detecting unilateral PA is often accompanied by lower sensitivity). As an example, Burrello et al [5] developed a 20-point score to predict unilateral PA, combining aldosterone concentration, serum potassium, and CT imaging results; a score greater than 12 had a specificity of 83% and sensitivity of 94% for unilateral PA, while a score greater than 16 had a higher specificity of 98% and lower sensitivity of 47%. Our choice experiment indicated that, at the base case, consumers preferred the algorithm when the accuracy of AVS and PET-CT was set at 90% or below.

The LCL analyses helped identify 2 potential distinct latent classes among participants, and this information enriched our understanding of the profile of participants who should be focused on to improve the subtyping uptake. This class included younger adults and men [22], who are less likely to engage with the health system. A recent systematic review highlighted the barriers men face in relation to their access to health care. Barriers identified included the “fear of diagnosis, treatment, or mortality,” reluctance and delays in seeking help, stoicism and self-reliance, symptoms minimization, poor health literacy, and low service knowledge [23]. A structural barrier identified was busy schedules [24], with some men reporting difficulty finding appointments for times they found convenient [23]. This was especially true for younger men. Improvements in diagnostic accuracy offset some of the negative effects of high out-of-pocket costs; policies that minimize out-of-pocket costs would be important in encouraging consumers to undergo the necessary procedures to reach an accurate diagnosis of surgically curable disease (with longer-term value for money) [2].

### Limitations

Patients both with lived experience and those who could potentially be future users of a health technology or clinical pathways are important in the use of DCE to explore preferences for such services as insights from both groups provide meaningful information. However, as PA is currently underdiagnosed in Australia, it is not feasible to recruit a group of respondents who are representative of individuals with PA in real life who have not yet undergone treatment. Given this constraint, we focused on hypertensive patients, for whom PA diagnosis and subsequent subtyping are highly relevant. However, we did not exclude from the study hypertensive participants who had been diagnosed with PA.

While our DCE scenario asks all hypertensive patients which subtyping procedure they would prefer, this is a simplification of what would happen in the clinical context in real life. Not all hypertensive patients would have PA, and not all PA patients will need to be or would want to be subtyped. To account for patient preferences, discussions with patients on subtyping probability are therefore important and should take place before a subtyping procedure is offered. Future studies should externally validate the stated preference findings from this study. Close to 17.3% of our participants indicated an understanding of what PA is and indicated they are currently being tested for it. This is likely higher than estimated for the general population, given rates of diagnosis of PA have been reported to be as low as 0.1% to 0.45% of patients with hypertension (based on survey data from 10 general practitioner clinics in Australia) [25]. This is likely due to chance sampling variability, as participants were recruited from an online general population panel. It is also possible that the survey title or description may have attracted individuals with an interest or experience in PA to participate. Nonetheless, sampling quotas were applied to ensure representativeness in terms of age, sex, and state of residence.

As it is likely that having experience changes preferences, we conducted a sensitivity analysis in which participants who had either received treatment or were currently in the process of receiving PA were dropped from the sample and obtained very similar results, but with a stronger preference for the predictive algorithm compared to the other 2 subtyping methods.

While we obtained patient input during the study design phase and provided information about PA, the rationale for subtyping, and its role in treatment decisions in the preamble to the DCE, we cannot be certain that all participants fully engaged with or comprehended the information to the extent necessary to appreciate the clinical implications of their choices. The DCE format requires a balance between comprehensiveness and cognitive burden, and as such, some complexity, particularly regarding the consequences of diagnostic misclassification, may not have been fully conveyed. For instance, participants may not have fully grasped that misclassification as unilateral PA could result in an unnecessary adrenalectomy. Additionally, responses may have been influenced by varying levels of health literacy or cultural beliefs, despite our efforts to provide clear and accessible background information. Nevertheless, the consistent preference patterns observed across a diverse sample suggest that key drivers of choice, such as invasiveness, cost, and waiting time, were broadly understood and reliably captured.

To help understand the effect of health literacy and cultural beliefs, future studies could collect information on cultural beliefs that are likely to affect health-related decision-making and control for this in subsequent analyses. In the case of low health literacy, providing information sheets well in advance of the survey to all potential respondents to ask any clarification questions could be a potential way forward. Providing the survey in relevant languages including in Easy English (simple, without unnecessary details for people with either limited English skills or who find it difficult to read and understand sentences in English) and including questions on prior health knowledge could also help control and account for the effect of low health literacy and cultural differences in subsequent analyses.

Additionally, as PET-CT is currently not in use for PA subtyping, its associated costs and wait times were obtained from other types of PET-CT scans (eg, AU$953 [26] to AU$1300 [27] for cancer screening) while accuracy was obtained from published studies [10-14].

Our attributes did not cover travel distance or logistics, which can affect choice in the real-life setting; for example, rural/remote areas are likely to have limited access to AVS or PET-CT. One can reasonably predict that the preference would be higher for predictive algorithms in these settings. That being said, our LCL analyses did not show a statistically significant effect of rurality/remoteness on the choice of subtyping services. Our survey was available only in the English language and limited to participants from Australia; multicultural communities may display different choices, hence additional DCEs in diverse languages in different countries would provide more generalizable information. There is also the possibility of selection bias. Not all invited participants would have agreed to participate in the survey as online panels typically involve the distribution of invitations via the research panel company. In our survey, roughly 262 invited participants did not participate in the survey, including 73 who did not click on the link sent. The average age of those opting out (either by not clicking the link or not consenting) was about 42 years. Of all the participants who clicked on the link, only 6% (with and without hypertension) did not consent to participate in the survey. Full details of participants excluded from the full sample are provided in Supplementary Fig. S2 [17].

Our study highlights a strong consumer preference for simpler, faster, and noninvasive PA subtyping strategies, even at the cost of a modest reduction in diagnostic accuracy. These findings demonstrate that many individuals with hypertension are willing to trade a small degree of precision for the benefits of lower cost, reduced waiting time, and fewer side effects. With the growing recognition of PA and an anticipated increase in screening and diagnosis, it is critical that subtyping strategies evolve in ways that reflect consumer preferences. Our results underscore the importance of continued research to optimize predictive algorithms and functional imaging, and to support the equitable implementation of alternative subtyping approaches that meet both clinical and consumer needs. Algorithms are not intended to replace AVS or PET-CT, but may play a role in guiding which patients are most likely to benefit from further testing and therefore serve as a highly accessible first step in the subtyping cascade.

## Data Availability

Some or all datasets generated during and/or analyzed during the current study are not publicly available but are available from the corresponding author on reasonable request.

## Abbreviations

AU$: Australian dollars
ASC: alternative specific constant
AVS: adrenal vein sampling
CI: confidence interval
CT: computed tomography
DCE: discrete choice experiment
LCL: latent class conditional logit
PA: primary aldosteronism
PET-CT: positron emission tomography–computed tomography
